# A single-cell genome reveals diplonemid-like ancestry of kinetoplastid mitochondrial gene structure

**DOI:** 10.1098/rstb.2019.0100

**Published:** 2019-10-07

**Authors:** Jeremy G. Wideman, Gordon Lax, Guy Leonard, David S. Milner, Raquel Rodríguez-Martínez, Alastair G. B. Simpson, Thomas A. Richards

**Affiliations:** 1 Living Systems Institute, University of Exeter, Stocker Road, Exeter EX4 4QD, UK; 2 Department of Biochemistry and Molecular Biology, Dalhousie University, Halifax, Nova Scotia, Canada B3H 4R2; 3 Department of Biology and Centre for Comparative Genomics and Evolutionary Bioinformatics, Dalhousie University, Halifax, Nova Scotia, Canada B3H 4R2; 4 Laboratorio de Complejidad Microbiana y Ecología Funcional, Instituto Antofagasta, Universidad de Antofagasta, Antofagasta, Chile

**Keywords:** diplonemids, kinetoplastids, mitochondrial genome, single-cell genomics, evolution

## Abstract

Euglenozoa comprises euglenids, kinetoplastids, and diplonemids, with each group exhibiting different and highly unusual mitochondrial genome organizations. Although they are sister groups, kinetoplastids and diplonemids have very distinct mitochondrial genome architectures, requiring widespread insertion/deletion RNA editing and extensive *trans*-splicing, respectively, in order to generate functional transcripts. The evolutionary history by which these differing processes arose remains unclear. Using single-cell genomics, followed by small sub unit ribosomal DNA and multigene phylogenies, we identified an isolated marine cell that branches on phylogenetic trees as a sister to known kinetoplastids. Analysis of single-cell amplified genomic material identified multiple mitochondrial genome contigs. These revealed a gene architecture resembling that of diplonemid mitochondria, with small fragments of genes encoded out of order and or on different contigs, indicating that these genes require extensive *trans*-splicing. Conversely, no requirement for kinetoplastid-like insertion/deletion RNA-editing was detected. Additionally, while we identified some proteins so far only found in kinetoplastids, we could not unequivocally identify mitochondrial RNA editing proteins. These data invite the hypothesis that extensive genome fragmentation and *trans*-splicing were the ancestral states for the kinetoplastid-diplonemid clade but were lost during the kinetoplastid radiation. This study demonstrates that single-cell approaches can successfully retrieve lineages that represent important new branches on the tree of life, and thus can illuminate major evolutionary and functional transitions in eukaryotes.

This article is part of a discussion meeting issue ‘Single cell ecology’.

## 1. Introduction

The Euglenozoa are a diverse group of protists well-known for their distinctive cellular features. They are flagellates with a (mostly) complete corset of microtubules supporting the cell membrane, a characteristic, elongated feeding apparatus (at least ancestrally), and usually two flagella, each with a different type of paraxonemal rod [1,2]. Euglenozoa are divided into three major groups: euglenids, kinetoplastids, and diplonemids [2,3]; a fourth group, symbiontids, is either sister to euglenids or a subgroup within them—see [4]. Euglenids include well-studied phototrophs and osmotrophs, and a wide diversity of phagotrophs that can be among the most abundant heterotrophic flagellates by biomass in some benthic biomes [4]. Kinetoplastids are well known for their importance as parasites, but also include common free-living phagotrophs [5]. Diplonemids have historically been far less studied, but have recently been shown to be one of the most diverse groups of microbial eukaryotes in the upper ocean water column [6,7]. A recent single-cell genome study linked to imaging revealed that marine diplonemids are morphologically diverse and have nuclear genomes with non-canonical introns, making it complicated to identify candidate open reading frames [8]. Kinetoplastids and diplonemids form a robustly supported clade to the exclusion of euglenids in many molecular phylogenies [9,10], and have recently been grouped into a taxon called Glycomonada [11] based on their shared characteristic of possessing glycosomes—derived peroxisomal homologues that house subsets of enzymes which catalyse glycolysis/gluconeogenesis, the pentose phosphate pathway, and lipid metabolism [12–15].

Euglenozoans have some of the most diverged mitochondrial genomes of all eukaryotes [16], which stands in contrast to their relatives, the jakobids, heteroloboseans and *Tsukubamonas* that usually have gene-rich, circular-mapping mitochondrial genomes [17–19]. Instead, the three major euglenozoan groups appear to have dramatically different mitochondrial genome architectures. The mitochondrial genome of *Euglena gracilis* comprises linear chromosomes containing single protein-coding genes which, unlike both diplonemid and kinetoplastid transcripts, are transcribed into mature messenger RNA (mRNA) without a need for editing [20]. Kinetoplastid mitochondrial genomes are more complicated [21]. Protein and ribosomal RNA (rRNA) genes are encoded on maxicircles approximately 20 kb in size; however, the information encoded on these maxicircles is often unrecognizable. It is only after substantial post-transcriptional editing by insertions and deletions of uracil nucleotides that the open reading frames of standard mitochondria-encoded proteins can be assembled. These edits are facilitated by guide RNAs, typically encoded on a separate class of DNA molecules, the minicircles (approx. 1 kb). Not to be outshone by their relatives, diplonemid mitochondrial genomes comprise a multitude of circular chromosomes (approx. 80+), many of which only encode small pieces of proteins [22]. In order to produce functional mitochondrial transcripts, RNAs transcribed from these ‘modules’ must be correctly *trans*-spliced, sometimes with intervening poly-U additions as well. Some substitution-type RNA-editing (A-to-I and C-to-U) also takes place [23]. The evolutionary transitions that occurred between the different euglenozoan mitochondrial genomic architectures remain unknown.

In order to understand these evolutionary transitions, additional deep-branching euglenozoans need to be examined. Single-cell genomic techniques have allowed greater access to genomic information from uncultured groups of organisms [24–26], including diplonemids [8]. Here, we use single-cell genomics to identify a novel sister taxon to known kinetoplastids. The genomic data from this cell includes putative mitochondrial contigs containing non-contiguous fragmented genes, whose transcripts presumably require diplonemid-like *trans*-splicing. These data suggest that the kinetoplast evolved from mitochondrial DNA with a gene architecture more similar to extant diplonemids, broadly consistent with previous proposals [27].

## 2. Methods

### (a) Single-cell genome sequencing

Single-cell amplified genome (SAG) sequencing was performed using a previously established pipeline, reported in detail here (https://dx.doi.org/10.17504/protocols.io.ywpfxdn). Briefly, heterotrophic flagellates in a marine water sample collected in Monterey Bay (36.6893° N; 122.384° W) were stained with Paclitaxel Oregon Green 488 Conjugate (Thermo Fisher), isolated with a BD Influx flow cytometer, and subjected to multiple displacement amplification (REPLI-g Single Cell Kit, Qiagen), and single-cell genome sequencing (Illumina HiSeq 2500, 250 bp paired-end library). One isolate (SAG D1), subsequently identified as a deep-branching euglenozoan, was chosen for further analysis. The original library preparation was re-sequenced (300 bp paired-end) to obtain a higher genome coverage which was then used for the analysis in this study. The SAG library was assembled using the automatic workflow available at https://zenodo.org/record/192677 and https://github.com/guyleonard/single_cell_workflow. Multiple assemblies were computed; either by using single libraries only or by combining the original sequencing effort with the resequencing library. Briefly, the Illumina HiSeq 250 bp paired-end read libraries were overlapped using bbmerge (https://jgi.doe.gov/data-and-tools/bbtools) in order to create ‘long’ reads. Those, together with the pairs that did not overlap, were subsequently quality- and adaptor-trimmed using the program Trim Galore! (https://www.bioinformatics.babraham.ac.uk/projects/trim_galore). The resulting *in silico* libraries were then assembled with SPAdes (v. 3.12.01) [28] using single-cell mode, the ‘careful’ option and with a combination of k-mers (21, 33, 55). Quality assessment of the resulting scaffolds was computed with the analysis software QUAST [29] and completeness profiles were generated using CEGMA and BUSCO [30,31]. A set of blobtools charts were also made with a combination of scaffolds, read mapping and megaBLAST hits to the NCBI nt database [32]. We also conducted blobtools including scaffolds of at least 1000 bp only using BLASTx and the NCBI nr database. These analyses failed to identify any consistent signal from a prokaryote genome, which rules out the presence of a prokaryotic endosymbiont in this cell sample, in contrast to reports for other euglenozoan taxa [33,34]. Additionally, Qualimap was run to provide reports of read mapping/coverage for this data [35].

### (b) Phylogenetic analyses (small sub unit)

A single small sub unit ribosomal DNA (SSU rDNA) contig (approx. 75% full length—deposited in NCBI's GenBank as MK578680) was extracted from the single-cell genome of SAG D1 using the *Diplonema papillatum* SSU rRNA gene (KF633466) as a BLAST query. The extracted SSU rDNA was queried against the NCBI nr database with BLASTn [36]. Based on these results, we constructed an alignment that contains an even sampling of currently sequenced groups of Euglenozoa. Additionally, the top three hits (ranked by identity) were included, resulting in a final Euglenozoa-wide dataset of 197 taxa. This dataset was aligned with MAFFT E-INS-i (v. 7.310) [37], checked manually with AliView (v. 1.17) [38], and masked with trimAl (v. 1.4; -st 0.001 -gt 0.83) [39] to exclude ambiguous sites (1125 sites retained). A maximum likelihood analysis was carried out with RAxML (v. 8.2.6) [40] under the GTR+Γ model with 20 random starting trees, and robustness assessed with 1000 bootstrap (BS) replicates. We additionally carried out a Bayesian analysis with MrBayes (v. 3.2.6) [41] under the GTR+Γ model, with duplicates running four chains for 5 000 000 generations each (default heating parameters), with trees sampled every 1000 generations and the first 25% discarded as burn-in. We confirmed convergence by assuring that potential scale reduction factor values approached 1.0.

### (c) Phylogenetic analyses (multigene)

A previously published pipeline for phylogenomic analyses using 351 conserved eukaryotic genes was used to extract relevant contigs from the SAG D1 single-cell genome [42,43] identifying 82 candidate conserved eukaryotic homologues present in the D1 assembly. Out of 82 extracted genes, a cut-off of greater than or equal to 40% site coverage was used to select a set of 30 genes. Genomes and transcriptomes of members of other discobid taxa were input into the same pipeline (electronic supplementary material, table S1) and used to assemble a preliminary dataset of 30 genes from 26 taxa. Each individual gene alignment was carried out with MAFFT L-INS-i (v. 7.407) [37], trimmed with BMGE (v. 1.0; -m BLOSUM62 -g 0.4) [44], and single-gene trees estimated for each with IQ-Tree (v. 1.5.5) [45] under the LG + C20 + F + Γ model and 1000 ultra-fast bootstraps (UFB) [46]. Each tree was checked for paralogous or contaminant sequences, as well as horizontal- or endosymbiotic gene transfers. Two genes were removed from further analysis because of several long-branching and/or potentially contaminant sequences, and a site coverage of less than 50% for SAG D1. Our final cleaned, trimmed dataset of 28 genes from 26 taxa was then concatenated, yielding a 4877 amino acid alignment, which was used to infer a final multigene phylogeny with IQ-Tree under the LG + C20 + F + Γ model, with robustness assessed with 200 non-parametric BS replicates as well as a 1000-replicate UFB approximation [46].

### (d) Mitochondrial genome analysis

Mitochondrial genome contigs were extracted from the SAG D1 single cell genome using *Andalucia godoyi*, *Diplonema papillatum*, and *Trypanosoma brucei* predicted mitochondrial proteins as tBLASTn queries. Contigs with putative mitochondria-encoded proteins were used as BLASTx queries of the NCBI nr database limited to the above-mentioned three eukaryotes, and also subjected to analysis by mfannot (http://megasun.bch.umontreal.ca/cgi-bin/mfannot/mfannotInterface.pl). Only contigs that were predicted by mfannot to have mitochondrial proteins encoded, or whose top hits were mitochondria-encoded proteins from other eukaryotes, were considered to be *bona fide* contigs derived from the SAG D1 mitochondrial genome. A total of eight mitochondrial contigs were identified, ranging from 774 to 7242 bp. Because diplonemid mitochondrial chromosomes share very similar non-coding regions [22], we used these mitochondrial contigs to search for other putative mitochondrial sequences and identified 16 contigs (greater than 500 bp) with similar (but not identical) regions. Owing to the large number of Cox1-encoding fragments identified, they were specifically chosen for comparison to conserved fragments from *Diplonema ambulator* and *D. papillatum*.

### (e) Phylogenetic analysis (Cox1)

To confirm that the Cox1-encoding fragments do not stem from contaminating (diplonemid) sequences in our assembly, we constructed a 19-taxon phylogeny of *cox1*, with a similar taxon sampling to our phylogenomic analysis. The dataset was aligned with ClustalO [47]; (default parameters), trimmed with bmge (v. 1.0; default parameters) to a final dataset of 402 amino acids. A phylogeny was estimated with IQ-Tree (v. 1.5.5) under the LG4M model with 500 non-parametric BS replicates.

### (f) Confirmation of SAG D1 mitochondrial genome architecture by polymerase chain reaction

To confirm the architecture of the 7242 bp contig from SAG D1, polymerase chain reaction (PCR) was performed using Q5 polymerase (New England Biolabs) with primers Nad5_F2 (5′-ATTTCACTCATCCGGTACTTACG-3′) and Nad8_R2 (5′-TGATAAGGCGAATGGAGGAC-3′; 2698 bp amplicon—see figure 3*a* for representation). Each 25 µl reaction contained 0.5 µM each primer, 200 µM dNTPs and 1 ng template DNA. Cycling conditions were 30 s at 98°C followed by 30 cycles of 10 s at 98°C; 20 s at 60°C; 2 min at 72°C, then a final extension of 2 min at 72°C. PCR products were purified (Promega Wizard SV Gel and PCR Clean-Up System), A-tailed using GoTaq G2 Flexi DNA polymerase (Promega) and cloned using a StrataClone PCR Cloning Kit (Agilent Technologies). Plasmids were then Sanger sequenced using M13F/R primers (MWG Eurofins).

### (g) U1 and intron identification

We searched for introns in all 28 candidate nuclear encoded genes used for the phylogenomic dataset described above. Three different contigs, encoding ATG2, H4, and D2HGDH were suspected to contain introns and were analysed using the NetAspGene v. 1.0 Server [48] at http://www.cbs.dtu.dk/services/NetAspGene/. Although this program was designed to predict canonical introns in *Aspergillus* species, it predicted convincing splice sites in these three SAG D1 contigs. A single 3′ splice not detected by NetAspGene was manually predicted based on homology. The U1 sequence previously identified in single cell genomic analyses of marine diplonemids [8] was used as a query to extract the putative U1 sequence from SAG D1. The secondary structure of U1 was inferred manually, and visualized using forna [49].

## 3. Results and discussion

### (a) Single-cell genomics identifies novel deep-branching lineage sister to kinetoplastids

As part of a large project of single-cell isolation from marine environments, we identified a cell-sample and subsequent SAG as belonging to a deep-branching euglenozoan (see https://dx.doi.org/10.17504/protocols.io.ywpfxdn for information on cell isolation protocol). This isolate—SAG D1—was chosen for re-sequencing to gain better genomic coverage for further investigation. A total of 39.2 Mbp of sequence was assembled, with 17.3 Mbp in contigs ≥ 1000 bp (N50 = 895 bp; with 9464 contigs under 1000 bp). The mean coverage was 41.6× with a standard deviation of 62.6×. The extremely high standard deviation probably indicates that the genome has some highly repetitive regions that are over-represented in the raw reads. The SAG D1 genome had a CEGMA completion of only 4% with partial proteins included in the analysis, but contigs with nearly full-length SSU and large sub unit (LSU) rDNA genes were identified. The low completeness is at least partially owing to the difficulties inherent in single-cell genome amplification and sequencing. Because of the incomplete nature of this SAG, we focused primarily on phylogenomic investigations, mitochondrial gene structure analysis, and presence of nuclear introns.

**Figure 1.**
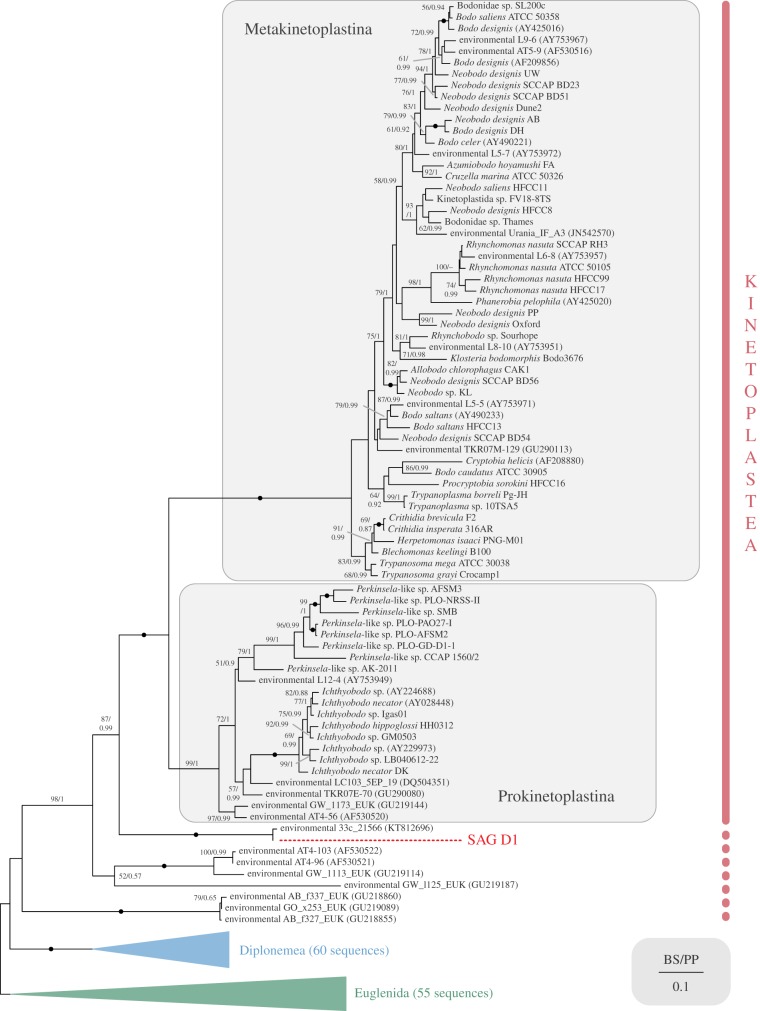
SSU rDNA phylogeny of Euglenozoa, showing SAG D1 as a sister to known kinetoplastids. Estimated under maximum likelihood GTR + Γ, with sequence of SAG D1 shown in red, and subgroups of Euglenozoa collapsed for clarity (for full tree see the electronic supplementary material, figure S1; Euglenida here includes Symbiontida). Bootstrap (BS) supports for each node are listed first, with posterior probabilities (PP) from Bayesian analysis second. Support values lower than 50% and 0.9 PP are not shown, and fully supported (100%/1 PP) nodes are denoted by a black circle.

Searching the NCBI nr database with an extracted SSU rDNA fragment of SAG D1, a single sequence 99% identical to 100 bp of our query was retrieved (environmental clone ‘33c-21566’, KT812696). Subsequently ranked BLAST hits were clearly related to either kinetoplastids or diplonemids. To determine the exact placement of this taxon, we reconstructed the phylogeny of euglenozoans based on nuclear SSU rDNA sequences (figure 1 and electronic supplementary material, figure S1). SAG D1 plus KT812696 formed a clade that branched robustly as sister to known kinetoplastids (i.e. to the maximally supported clade consisting of Metakinetoplastina and Prokinetoplastina).

**Figure 2.**
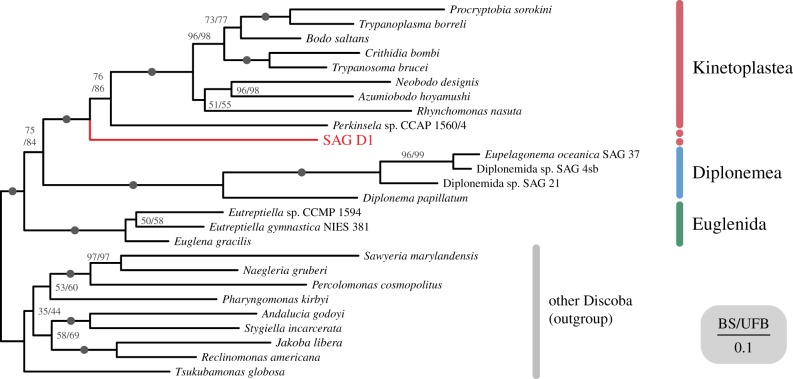
Phylogenomic analysis confirms SAG D1 as a sister to kinetoplastids. Twenty eight-gene maximum likelihood phylogenetic tree of Discoba with SAG D1 in red, estimated under LG+C20 + F + *Γ* model. First numbers on nodes show bootstrap support values derived from 200 non-parametric bootstraps, second numbers show support from 1000 ultra-fast bootstraps, with full support from both phylogenetic methods denoted by a black circle.

The blobplot generated from the SAG D1 genome (electronic supplementary material, figure S2) was used to assess possible sources of identifiable sequence contamination. This demonstrated no substantial signal of prokaryotic, viral or eukaryotic contamination, suggesting that the SAG is suitable for phylogenomic analyses. A phylogeny inferred from 28 conserved genes in 26 taxa from Discoba mirrored the result of the SSU rDNA phylogenetic analysis (figure 2), placing SAG D1 as the closest relative of known kinetoplastids. The relationship between SAG D1 and known kinetoplastids received full non-parametric BS and UFB support, while sisterhood of Metakinetoplastina and Prokinetoplastina (represented by *Perkinsela* sp.) and was supported by 76% BS/86% UFB (Metakinetoplastina was fully supported). Diplonemids and euglenids were each recovered with full support, as was the bipartition between Euglenozoa and the other members of Discoba (figure 2).

### (b) Mitochondrial trans-splicing in the ancestor of diplonemids and kinetoplastids?

Using the predicted mitochondrial protein sequences from the jakobid *A. godoyi*, the diplonemid *D. papillatum*, and the kinetoplastid *T. brucei*, we searched for mitochondrial contigs encoded in the partial genome of SAG D1. We identified eight *bona fide* mitochondrial contigs containing fragments of mitochondrial protein coding genes and an additional 16 putative mitochondrial contigs with regions highly similar to short (approx. 100 bp) non-coding regions of one or more *bona fide* contigs. These contigs bear no obvious resemblance to any sequenced maxi-circle kinetoplastid DNA nor to any sequenced diplonemid mitochondrial chromosomes by BLASTn searches.

**Figure 3.**
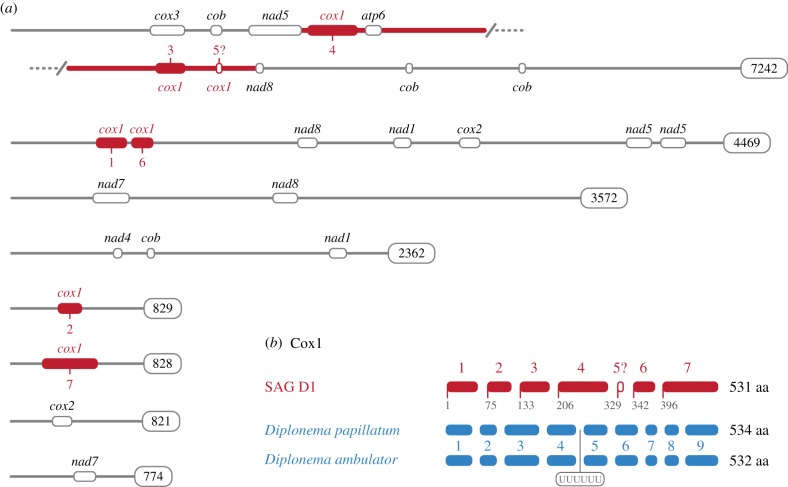
SAG D1 has a diplonemid-like mitochondrial genome architecture. (*a*) Eight contigs from SAG D1 contained modules encoding fragments of mitochondrial proteins. Verified mitochondrial contigs were extracted from assembled SAG D1 and analysed for the presence of proteins normally encoded by euglenozoan mitochondrial genomes. The bold red line in the first fragment (ranging from *nad5* to *nad8*) denotes a fragment that was also confirmed by PCR and Sanger sequencing. (*b*) SAG D1 Cox1 requires *trans*-splicing but not RNA editing. Several contigs contained Cox1 fragments which could be manually assembled into a contiguous transcript. The ‘?’ mark denotes a possible short gene module which a small extension of module 4 would render unnecessary. Note that *Diplonema* species have a 6× Uracil (UUUUUU) insertion between their modules 4 and 5.

To confirm that the assembled sequence of the largest 7242 bp mitochondrial contig was not due to assembly errors, we PCR-amplified and Sanger-sequenced a 2698 bp fragment from the middle of the contig stretching from the *nad5* module through *atp6*, 3 *cox1* modules to the *nad8* module (figure 3*a*). This confirms the open reading frame order and demonstrates that *trans*-splicing must be involved to generate a viable mRNA if this represents a true fragment of the D1 mitochondrial gene (discussed further below). From these results, we infer that *trans*-splicing is present deep within the kinetoplastid lineage, however, in the absence of RNA sequencing data it is not possible to exclude that D1 mitochondrial transcripts are also subject to some form of RNA-editing.

Organellar genomes differ from their respective nuclear genomes by GC content, tetranucleotide frequency, and relative abundance. Because mitochondrial genomes are usually present in much higher numbers than nuclear genomes, they are often over-represented in sequencing-based studies. Consistent with this, we found that the mitochondrial contigs had a higher-than-average coverage (196×) compared to the SAG assembly as a whole (41.6×) as well as the contigs encoding the 28 genes used for our nuclear gene phylogenomic analysis (91.4×). Additionally, the mitochondrial contigs had an average GC content of 36.1% compared to 45.7% for the SAG D1 genome assembly as a whole and 48.0% for the contigs used for phylogenomic analysis. Furthermore, tetranucleotide frequencies suggest a clear difference between the two sets of contigs (electronic supplementary material, figure S3). For this we created an ‘ESOM’ (a type of map based on the underlying distance of—in this case—tetranucleotide frequencies, as indicated in [50] and https://github.com/tetramerFreqs/Binning). As a control, we ‘spiked in’ a viral genome (*Escherichia* virus T4—NC_000866.4). The map shows a clear split between the three sets of contigs: green—T4 virus, orange—phylogenomic contigs and blue—mitochondrial contigs. The variation in tetranucleotide frequencies, lower GC content and specifically the higher coverage demonstrate that these sequences are generated from a different genomic template, consistent with the hypothesis that they are mitochondrial contigs and not part of the nuclear genome.

Also, consistent with the hypothesis that these are *bona fide* mitochondrial genome sequences and are not nuclear mitochondrial DNA segment (NUMT) sequences, we find no evidence of stop codon mutations within the six to seven identified putative *cox1*, which would be typical of pseudogenization often associated with formation of NUMTs. We cannot directly exclude that the putative mitochondrial contigs were derived from NUMTs and that the mitochondrial gene module orders identified were the product of in-nuclear re-arrangements. Nonetheless, we think this alternative explanation is unlikely because it suggests that rearrangements would have occurred at a rate faster than either stop codon mutations or changes in the GC and tetranucleotide biases during the transfer and formation of the candidate NUMTs. Alternatively, the putative mitochondrial contigs identified could be very recent NUMTs that have undergone very little sequence change, even if this is so, this would still provide evidence of the non-contiguous and scrambled gene module ordering of the *cox1* mitochondrial gene in the D1 kinetoplastid.

We could not identify rRNA or transfer RNA (tRNA) genes in any of the mitochondrial contigs. The lack of tRNA genes is anticipated, as the common ancestor of euglenozoans probably lacked mitochondria-encoded tRNAs, which are instead encoded in the nuclear genome and imported into mitochondria [16]. SSU and LSU rRNA genes could not be identified, probably because they were not recovered in the SAG or because euglenozoan mitochondrial rRNAs are extremely divergent and much smaller than those in other eukaryotes [16]; for example the *Diplonema papillatum* mitochondrial SSU rRNA is the shortest identified to date at 366 bp [51], and it is unclear whether full-length euglenid mitochondrial rRNAs have been identified [20,52].

Similar to diplonemid mitochondrial genomes, the identified mitochondrial contigs only encoded small protein coding fragments (up to approx. 135 amino acids long). Some contigs only contained one such gene ‘module’, whereas the largest contig (7242 bp) encoded 10 different modules, and the second-largest (4469 bp) contig encoded seven (figure 3*a*). Unlike the mitochondrial chromosomes in the best-studied diplonemid, *Diplonema papillatum*, the contigs identified here only have very short regions of high similarity—usually near their very ends—and are instead largely made up of a unique sequence. Thus, our data on SAG D1 mitochondrial chromosomes are not consistent with the ‘cassette’ structure seen in diplonemids [22].

In all, modules corresponding to parts of *atp6*; *nad1*, *4*, *5*, *7*, *8*; *cox1*, *2*, *3*; and *cob* were identified, all of which are genes known to be encoded in both diplonemid and kinetoplastid mitochondrial genomes (figure 3*a*; [22]). Most of the encoded fragments are isolated tracts of amino acid coding sequence that could not be paired with other fragments. The exception was the Cox1 protein, for which six or seven gene modules were identified that putatively encode a contiguous mature mRNA (see below). These are present on four different contigs, and clearly in a scrambled order (e.g. modules 1 and 6 are nearby on one contig, while modules 4, 3 and 5 are on another, in that order; figure 3*a*). This implies that *trans*-splicing would be necessary to assemble the mature transcript. Given our strict assembly criteria, PCR and resequencing, and the fact that these mitochondrial contigs are among the best sampled in terms of sequencing reads, this represents strong support for a fragmented gene module structure for *cox1*.

Cox1 protein fragments from SAG D1 and the conserved fragments from representatives of Diplonemidae (the best studied subgroup of diplonemids) were manually compared to determine if splice sites are conserved between diplonemids and our novel lineage (figure 3*b*). Similar to *Diplonema* species Cox1 fragments, no post-transcriptional substitution editing appeared to be necessary to produce a translatable mRNA. *Diplonema papillatum* and *D. ambulator* (Diplonemidae) share conserved modules and splice sites; whereas *Hemistasia phaeocysticola* (Hemistasidae)—in addition to the conserved sites—has further non-conserved splice sites [51,53]. SAG D1 shares at most only one of the ‘conserved’ splice sites (between modules 4 and 5). However, module 5 is the shortest of all the predicted internal modules, and module 4 could be extended through a poorly conserved region, eliminating the requirement for putative module 5 entirely (marked as ‘?’ in figure 3). The fact that none of the SAG D1 modules appear to be directly homologous to those of diplonemids suggests either that a mechanism for module remodelling exists, or the module architecture itself had multiple origins.

Most mitochondrial transcripts in *D. papillatum* undergo uracil-appendage editing at, on average, one linkage site between modules [22]. The *cox1* transcript of *D. papillatum* has a single 6× uracil appendage between modules 4 and 5. The homologous region of SAG D1 would lie in the middle of module 4. While this means that uracil-appendage editing at this site is not conserved, it does not rule out the possibility that it occurs at non-homologous sites. Only with mRNA sequencing data will we be able to more fully understand RNA editing and *trans*-splicing in this lineage.

Our results indicate that the putative mitochondrial contigs are from a different genomic source and we therefore sought to confirm that they are from the same cell as SAG D1 nuclear contigs and not from a contaminant (e.g. a diplonemid). We therefore performed a phylogenetic reconstruction using the assembled Cox1 protein with a similar taxon sampling as for our nuclear protein phylogenomic analysis (electronic supplementary material, figure S4). As in figure 2, SAG D1 Cox1 branched sister to all known kinetoplastids with nearly full BS support. These results further confirm that SAG D1 comes from a lineage sister to known kinetoplastids that retains ancestral diplonemid-like mitochondrial gene features.

### (c) Kinetoplastid-specific proteins present in SAG D1

Because it is unclear which molecular characteristics are shared by all euglenozoans, versus which are group-specific, we searched for putatively euglenozoan-specific glycosomal membrane, mitochondrial ribosomal and membrane proteins, as well as components of the kinetoplastid RNA-editing machinery (accessions were obtained from [54]). Although the SAG D1 genome is rather fragmented, we were able to identify contigs encoding a small number of partial kinetoplastid-like orthologues using a reciprocal-best-hit method (electronic supplementary material, table S2). These included six mito-ribosomal, nine mitochondrial membrane, and three peroxisomal membrane proteins. Two homologues of proteins involved in RNA editing in *T. brucei* were also identified; however, several RNA editing proteins were also identified encoded in the genome of *E. gracilis* [52] suggesting that these genes were co-opted for their function in kinetoplastids. Thus, while their presence is not evidence of RNA-editing functions, we cannot rule out the possibility that kinetoplastid-like mitochondrial RNA editing occurs in this lineage. In total, six of the other identified proteins are probably euglenozoan-specific (e.g. four lineage-specific ribosomal proteins and two mitochondrial membrane proteins), as they have not been identified in other eukaryotes; however, it is unclear which components are kinetoplastid-specific. The presence of kinetoplastid-like ribosomal proteins in both *E. gracilis* and SAG D1 suggests that the shift from RNA- to protein-based mitochondrial ribosomal architecture occurred early in euglenozoan evolution [55]. More euglenid and diplonemid genomes are necessary for a better understanding of the molecular changes that have accompanied the major changes observed in euglenozoan mitochondrial genome and ribosomal architectures.

### (d) Canonical introns in SAG D1

**Figure 4.**
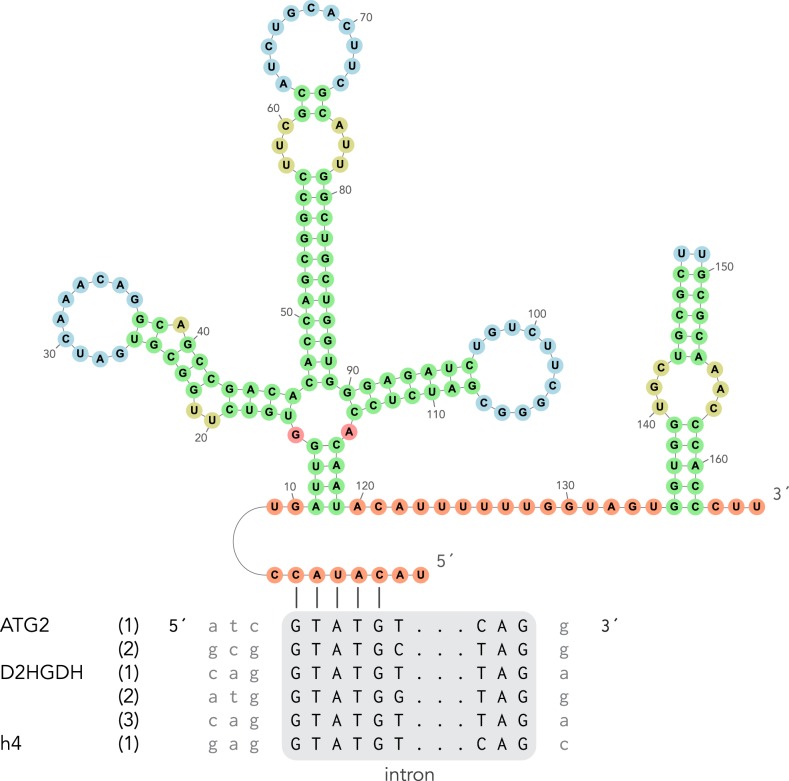
Inferred secondary structure of the U1 spliceosomal RNA of SAG D1, and inferred binding of its active site to identified canonical introns in our assembly (six putative splicing sites in three genes). Number of nucleotides are labelled in the secondary structure.

Kinetoplastid nuclear genomes are extremely intron-poor, with *T. brucei* having only two introns and *Perkinsela* possibly none at all [56,57]. Conversely, diplonemids are known to have many more canonical introns [58], and single-cell genomic analyses revealed that they also contain many non-canonical introns [8]. Of the 30 partial genes used in the phylogenomic analysis, we were unable to identify non-canonical introns, but found three genes with canonical introns (figure 4). We used the U1 sequence from [8] as a query to identify a candidate U1 sequence in the SAG D1 genome which can bind the identified canonical 5′ splice sites (figure 4). These findings indicate that the ancestral kinetoplastid probably contained canonical introns and possibly many more than detected in previously studied kinetoplastids.

## 4. Conclusion

Our analyses demonstrated that targeted single-cell genomics can be useful as a means for capturing unsampled eukaryotic diversity that is pivotal in tracing major evolutionary and cellular functional transitions in eukaryotic microbes. Multiple phylogenetic analyses robustly placed SAG D1 in a sister group to known kinetoplastids, allowing us to infer some ancestral features of the kinetoplastid lineage. In particular, because SAG D1 has highly fragmented genes similar to those of diplonemids, it is reasonable to hypothesize that this fragmentation represents the ancestral state for the diplonemid–kinetoplastid clade (Glycomonada) as suggested previously [27]. If so, the complex genomic structure and RNA editing of kinetoplastid mitochondria evolved from a more diplonemid-like ancestral state that relied on *trans*-splicing to assemble mature transcripts. This hypothesis would require that kinetoplastids re-evolved defragmented protein-coding mitochondrial genes, which seems implausible at first glance. A possible mechanism, however, would be the incorporation of reverse-transcriptase mRNA-derived DNA sequences into the mitochondrial chromosomes. This phenomenon probably explains the concurrent loss of numerous editing positions from individual mitochondrial genes in kinetoplastids, albeit facilitated by homologous recombination in that case [59].

Clearly, further work is merited on the lineage represented by SAG D1. Further SAG data would be, however, of limited value. Instead, a genomic plus transcriptomic approach would be extremely valuable, for example, to support examinations of nuclear gene content and structure as well as *trans*-splicing and detection of editing in mitochondrial mRNAs. We therefore call for a concerted effort to develop combined genome and transcriptome single-cell methodologies (e.g. [60]) specifically for environmental sequences. The SAG D1 SSU rDNA sequence may also be valuable to screen large numbers of isolated single cells and/or identify any sample types in which this lineage is abundant, and from which isolation of cells for cultivation could be attempted.

## Supplementary Material

Fig. S1 - SSU_full_supplements_v2.pdf

## Supplementary Material

Fig. S2 - Blob.pdf

## Supplementary Material

Fig. S3 - ESOM.pdf

## Supplementary Material

Fig. S4 - cox1_Discoba_v1.pdf

## Supplementary Material

Table S1

## Supplementary Material

Table S2

## Data accessibility

Raw reads of SAG D1: GenBank accession PRJNA379597. Extracted SSU rDNA fragment of SAG D1: GenBank MK578680. Assembly, phylogenetic data including alignments and single gene trees: doi:10.6084/m9.figshare.c.4427381.
