# Supplementary figures and images for: A single-cell genome reveals diplonemid-like ancestry of kinetoplastid mitochondrial gene structure
Source: Philos Trans R Soc Lond B Biol Sci. 2019 Oct 7;374(1786):20190100. doi: 10.1098/rstb.2019.0100 (PMC6792441; doi:10.1098/rstb.2019.0100)

# Metakinetoplastina

KINETOPLASTEA

# Prokinetoplastina

DIPLOMEA

EUGLENIDA

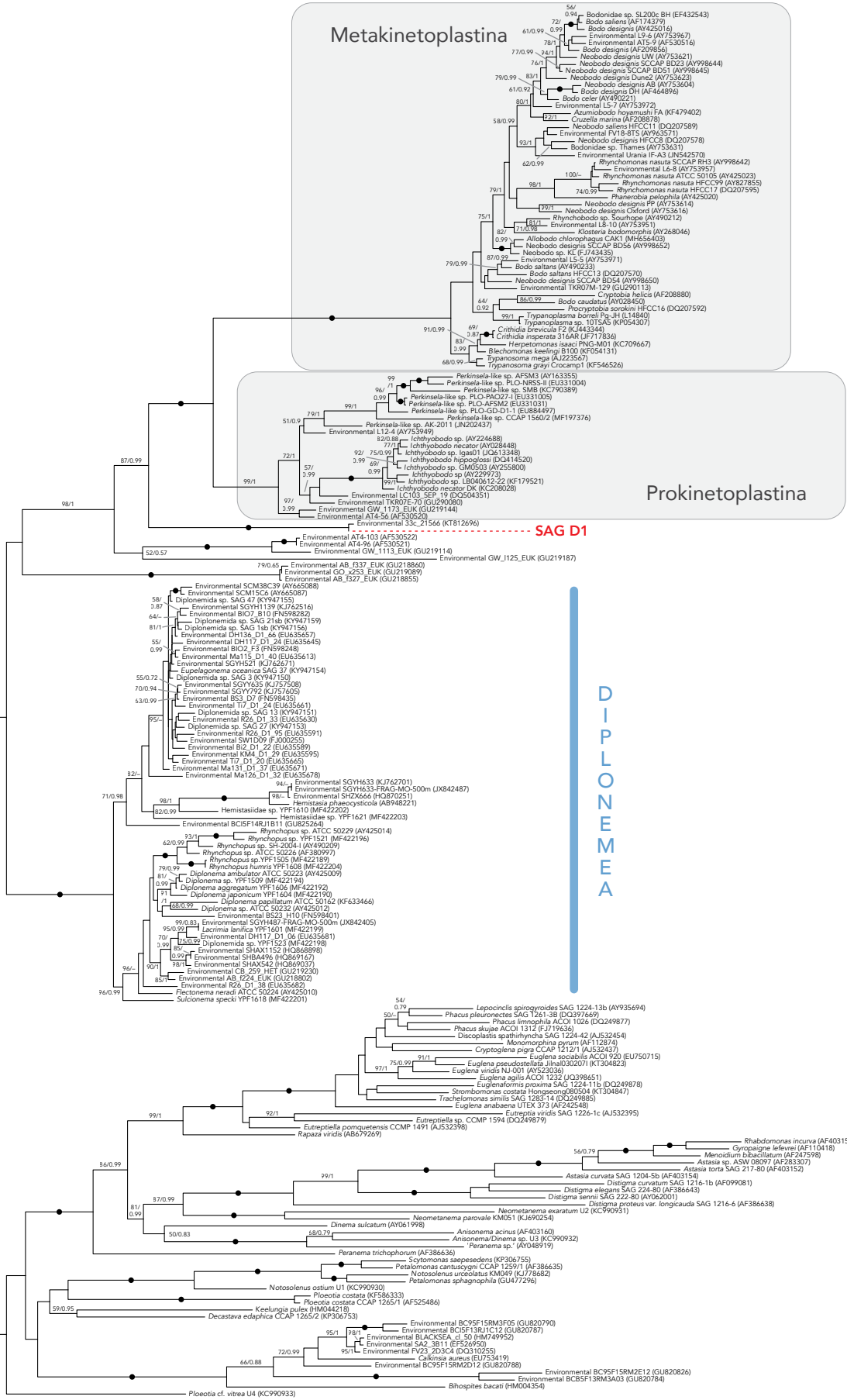

Supplement: Fig. S1 - SSU_full_supplements_v2.pdf [file rstb20190100supp1.pdf]

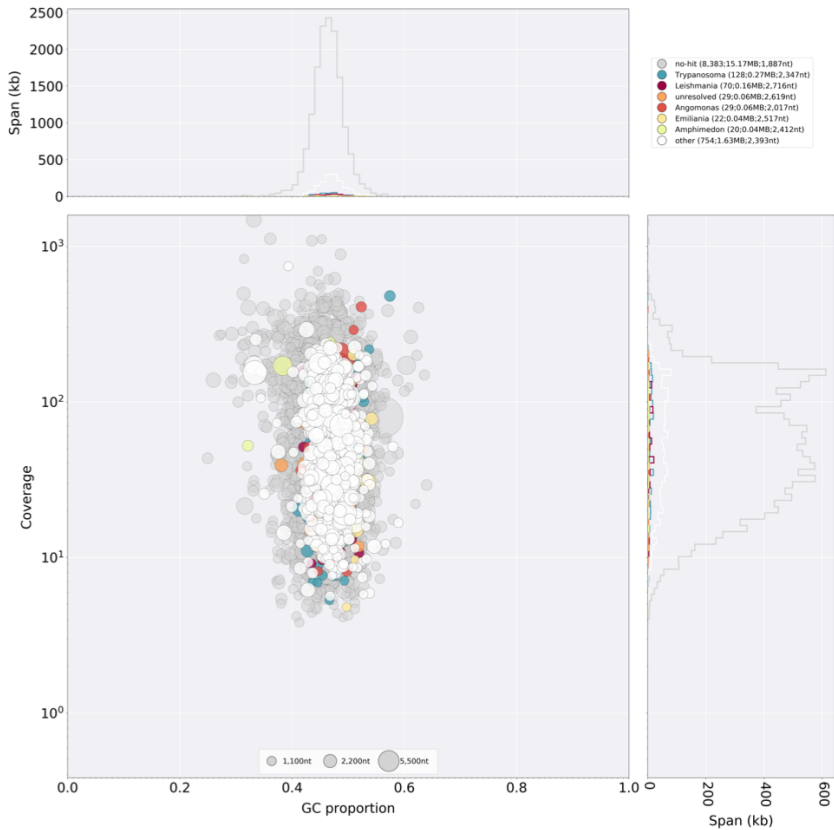

Supplement: Fig. S2 - Blob.pdf [file rstb20190100supp2.pdf]

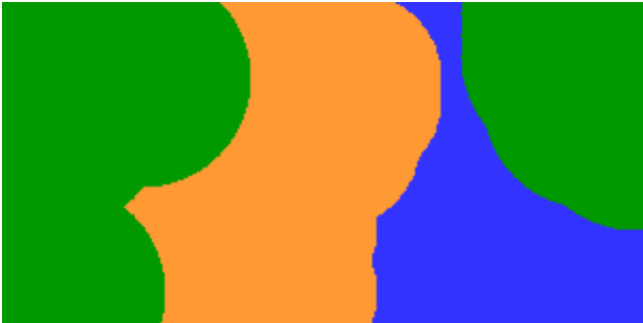

Supplement: Fig. S3 - ESOM.pdf [file rstb20190100supp3.pdf]

cox1

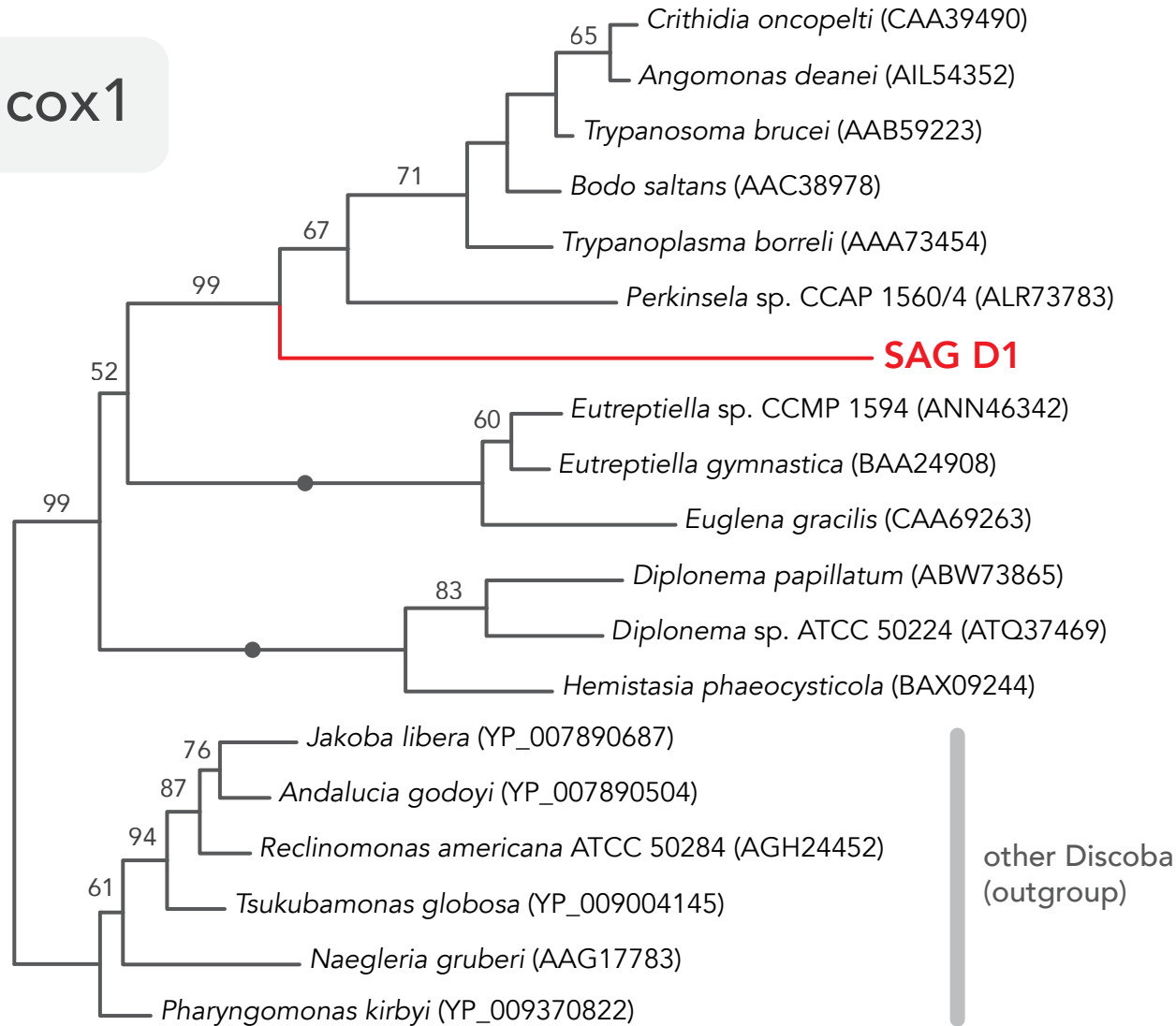

Kinetoplastea

Euglenida

Diplonemea

other Discoba  
(outgroup)

BS

0.5

Supplement: Fig. S4 - cox1_Discoba_v1.pdf [file rstb20190100supp4.pdf]
